# Functional mobility and physical fitness are improved through a multicomponent training program in institutionalized older adults

**DOI:** 10.1007/s11357-023-00877-4

**Published:** 2023-07-26

**Authors:** Sergio López-López, Vanesa Abuín-Porras, Luis A. Berlanga, Michelle Martos-Duarte, Luis Perea-Unceta, Carlos Romero-Morales, Helios Pareja-Galeano

**Affiliations:** 1 Faculty of Sport Sciences, Universidad Europea de Madrid, Madrid, Spain; 2 Department of Physical Activity and Sport, Centro de Estudios Universitarios Cardenal Spínola CEU, Seville, Spain; 3 Faculty of Health Sciences, Universidad Francisco de Vitoria, Madrid, Spain; 4 Department of Physical Education, Sport and Human Movement, Universidad Autónoma de Madrid, Madrid, Spain

**Keywords:** Aerobic exercise, Elderly people, Independence, Nursing home, Strength training

## Abstract

Physical exercise has demonstrated its effectiveness in the management of the deleterious process of aging. However, it is less studied in institutionalized elderly people. This investigation aims to clarify the benefits of a multicomponent training program in institutionalized older adults. A randomized controlled trial was conducted with institutionalized older adults (≥ 70 years old). Intervention group (IG; *N* = 18) were submitted to a multicomponent training program based on muscle power training and interval endurance exercise, 2 times/week for 12 weeks. Control group (CG; *N* = 16) continued their usual mobility exercises. Independence was estimated with the Barthel index, and physical fitness and functional mobility were evaluated by the Short Physical Performance Battery (SPPB), the Timed Up and Go (TUG) test, the 6-min Walking Test (6’WT), the 10-Meter Walking Test (10MWT), hand grip strength dynamometry, and lower limb muscle strength and power. The IG improved, compared with the CG, in TUG scores in -7.43 s (95% IC: 3.28, 11.59; *p* < 0.001); in 10MWT scores in -5.19 s (95% IC: 1.41, 8.97; *p* = 0.004) and -4.43 s (95% IC: 1.14, 7.73; *p* = 0.002), 6’WT scores in + 54.54 m (95% IC: 30.24, 78.84; *p* < 0.001); and SPPB in + 2.74 points (95% IC: 2.10, 3.37; *p* < 0.001). Maximum muscle power and maximum strength did not show statistically significant differences. The multicomponent training program based on muscle power and interval endurance exercise was shown to be safe, well tolerated and effective for the improvement of functional mobility and physical fitness, but not for independence in institutionalized older adults.

## Introduction

The world's population is aging, and a large part of the world's nations are experiencing an increase in the number and proportion of older adults [1]. Aging due to longer life expectancy is not necessarily related to a better quality of life during this increased survival period. In fact, there is a gap of approximately 9 years (7 for men and 11 for women) between life expectancy in good health and life expectancy. This means that the population will live for years with illness, disability and dependence [2]. A percentage of this elderly population, at some point, will plausibly be institutionalized in nursing homes.

Sarcopenia and frailty are two of the main characteristics found among institutionalized older adults. The progressive loss of lean mass and the consequent age-associated decrease in muscle strength are mainly due to the aging process and are considered the primary cause of sarcopenia. However, secondary sarcopenia is associated with factors commonly present in socio-health institutions where older adults reside, such as, lack of physical activity or a sedentary lifestyle, prolonged bed rest, presence of chronic diseases, multimorbidity, polypharmacy and nutritional deficiencies [3–6]. The frail elderly are those at greatest risk of developing a new disability. Frailty syndrome is associated with age and is characterized by a progressive loss of functional reserve [7].

Although the concept of frailty is widely recognized, there is a lack of consensus on the clinical and operational definition of the term [8–11]. This geriatric syndrome is described as the state of progressive physiological vulnerability to a stressor (e.g., acute illness, injury, surgery, disease, changes in medication) as a consequence of the accumulated deterioration in the various physiological systems over time, such that frail patients have an increasingly reduced capacity to recover their previous state of health once they have been exposed to physiological stress.

Regular physical activity is considered a protective factor for the prevention and management of many pathologies, such as cardiovascular disease, obesity, type 2 diabetes, colon and breast cancer [12–14], as well as for preventing premature mortality [15, 16]. Physical activity also grants mental health benefits [17, 18], delaying the onset of dementia and cognitive impairment [19, 20] and helping to maintain overall wellbeing [21]. Scientific evidence has established that there are additional benefits when combining different physical activity modalities (i.e. multicomponent training) with specific goals associated with improving physical fitness, such as strength, power, walking speed or balance [22]. Physical exercise, that develops muscle power, has been shown to be effective and safe in improving strength and muscle mass as well as functional capacity even in institutionalized nonagenarians [23, 24]. Balance in the elderly is generally impaired and is one of the factors contributing to the risk of falls. Strength exercise has shown to be effective in improving this component in elderly people living in nursing homes [25]. Moreover, aerobic resistance exercise has been shown to improve oxygen consumption capacity in people over 65 years of age [26].

Multicomponent physical exercise programs, and especially strength training, are established as the most effective strategies to delay the loss of strength and functional capacity [27, 28], disability and adverse events such as hospitalization. Likewise, they have demonstrated their usefulness in other areas related to frailty such as, fall risk, cognitive impairment and depression [29, 30]. This research work aims to clarify the effectiveness of a multicomponent training program in institutionalized elderly people on physical fitness, functional mobility and independence.

## Methods

### Study design

A prospective, longitudinal, randomized controlled trial was conducted following the recommendations and criteria of Consolidated Standards of Reporting Trials (CONSORT) [31]. The intervention was carried out at Albertia Elderly Care Center (Madrid, Spain).

### Ethical considerations

Prior to the performance of any procedure, the research report was approved by the Ethics Committee of Hospital Universitario la Princesa (Madrid, Spain). Compliance with the Declaration of Helsinki, [32] was guaranteed in this study. Participants read and signed the informed consent form before the intervention.

### Participants

Thirty-four participants were enrolled in the nursing home and randomly allocated using Microsoft Excel® software to the intervention group (IG; *N* = 18) or the control group (CG; *N* = 16). In the randomization procedure, each participant was allocated to either the control or the experimental group using a process implemented in Microsoft Excel. The RAND function was used to generate a random number for each participant. Based on the generated random number, participants were subsequently allocated to the control or experimental group.Participants’ inclusion criteria included: subjects (men or women) aged ≥ 70 years; frailty according to SPRINTT criteria (SPPB ≥ 3 & ≤ 9) [33]; ability to walk with or without technical aids; Barthel index ≥ 50; ability to communicate; ability to understand and sign the informed consent form. Exclusion criteria included: terminal illness; myocardial infarction in the last 3 months; unstable cardiovascular disease; fracture of extremities in the last 3 months; and severe dementia (GDS 7, Reisberg global deterioration scale). Elimination criteria during the study were: dropout from the study, absence of the patient at any evaluation, or a noncompliance rate < 50%.

### Sample size calculation

Employing G*power 3.1.9.2 software**,** sample size was calculated using the SPPB score, considering a 1-point difference as clinically relevant and taking the variability of this measure from previous studies in similar populations [34] (dt = 1.5 units) an α error of 0.05 was established, and a power of 0.80 with a 2-tailed hypothesis. A total sample of 32 individuals was estimated. Thirty-four participants were finally enrolled in prevention of study losses (women *n* = 25, men *n* = 9).

### Procedure

The interventions for the IG and CG were applied for 12 weeks, with a total of 32 sessions at a rate of 2 sessions per week. Each session lasted approximately 45 min and there were at least 48 h between sessions. The participants assigned to the IG were submitted to a multicomponent training program in which each bout of training began with a 5-min activation period, consisting of walking at the usual walking speed (obtained during the evaluation of the SPPB test battery) on a treadmill at 1% inclination (Treadmill Run 100, Domyos, Decathlon, France). The patients then performed two resistance exercises aimed at improving lower limb muscle power: leg press (Bodytone Evolution E59, Murcia, Spain) and plantar flexion exercise in standing using steps. For the leg press exercise, 3 to 4 sets of 8 to 15 repetitions were performed at an intensity between 30 to 60% of maximal strength (F0), with 1 min rest between sets. For plantar flexion, patients performed 3 sets of 4 to 12 repetitions with their body weight using one or both legs, with 1 min of recovery between sets. After that, participants performed an aerobic and intervallic exercise protocol on a treadmill, consisting in an 8–10 min program with 6–10 intervals, reaching ratios of 1:3 (30 s at maximum walking speed and 90 s at 50% of normal walking speed, and so on). In accordance with the principle of progression and individualization, the first two weeks were conceived as conditioning and the volume and intensity of the strength and aerobic exercises were adjusted. Finally, the program ended with 5 min of low active mobility.

The CG performed the nursing home’s usual care exercises program based on active mobility of most of the articular groups of the extremities during the 12 weeks of intervention.

### Outcome measures

The Timed Up and Go Test (TUG), is a functional mobility and balance test, particularly indicated for the evaluation of fall risk in elderly people [35]. The Short Physical Performance Battery (SPPB) [36] which consists of a balance test, a usual gait speed (UGS) test and a chair stand test was used for the evaluation of lower limb physical function. The 10-m walking test (10MWT) was employed for the evaluation of gait speed [37]. The test consists of measuring the time required to walk 10 m autonomously at the maximum possible speed for the patient. Functional capacity through stimulation of the cardiovascular system was measured through the 6-min walk test (6'WT) [38], which consisted of measuring the total distance covered during 6 min. Apart from the recording of the distance covered, blood pressure, heart rate and oxygen saturation were evaluated before and after the test [39]. The force-velocity profile (F-V) and muscle power were evaluated following the protocol of Alcazar et al. [40]. During the evaluation of the F-V profile and muscle power, patients performed 2–3 sets of 1 repetition with load increments until reaching an 8–10 on a perceived exertion scale. The rests between sets were 1–2 min, depending on the average speed achieved during the previously evaluated set. Strength and velocity were evaluated by a linear position transducer (Chronojump Boscosystem, Kit encoder lineal, Spain) during the concentric phase of the knee and hip extension movement in the leg press. The force and the highest average velocity of each load increment of each repetition were recorded. Any load that was not executed at maximum velocity was discarded, obtaining the F-V profile with the remaining valid measurements. The Barthel Index measures the person's ability to perform 10 activities of daily living, obtaining a quantitative estimate of the subject's degree of dependence.

### Statistical analysis

SPSS 23.0 software (IBM SPSS Statistics, Armonk-NY; IBM-Corp) was employed for the statistical analysis. Shapiro–Wilks’ test was used to assess the normality of the data distribution. Homogeneity of variance was assessed through Levene’s test. Effects of intra-subject (pre and post) and inter-subject (treatment groups) values were analyzed with a two-way analysis of variance (ANOVA) for repeated measures, and ηp2 values were considered for the effect size. The level of significance was set at *p* < 0.05 with an α error of 0.05 (95% confidence interval) and a desired power of 80% (β error of 0.2). For repeated measures ANOVAs, the potential influence of two covariates on the obtained measures was also analyzed: the percentage of compliance with the training sessions and the number of events for each participant.

## Results

Sociodemographic data and baseline measures showed no statistically significant differences in both groups at baseline, as well as moderator variables (number of adverse events and compliance) at the end of the intervention (Tables 1 and 2).

**Table 1:** Sociodemographic data, baseline measurements and moderator variables (Compliance and number of adverse events)

	CG (*n* = 16)		IG (*n* = 18)		Differences
	M	SD	M	SD	T-test (P)
Age (years)	86.19	5.53	85.78	6.79	0.849
Height (cm)	154.75	5.65	154.50	12.90	0.437
Weight (Kg)	63.21	13.19	27.04	8.05	0.512
BMI (Kg/m2)	26.31	4.70	63.95	3.94	0.605
Compliance (%)*	90.88	7.14	77.11	20.05	0.069
Events (nº)**	0.56	0.96	0.44	0.62	0.919

*CG*: control group; *IG*: intervention group; *M*: median; *SD*: standard deviation; *: calculated as a percentage of the total of 48 sessions; **: number of events (falls, hospitalization, exacerbation of previous illness) during the intervention

**Table 2:** Demographic data, baseline measurements compliance and events by sex

	Control				Experimental			
	Female *n* = 12		Male *n* = 4		Female *n* = 13		Male *n* = 5	
	M	DS	M	DS	M	DS	M	DS
Age (years)	86.50	5.58	85.25	6.08	86.92	7.39	82.80	4.09
Height (cm)	153.17	4.55	159.50	6.61	148.46	8.33	170.20	8.53
Weight (Kg)	58.87	10.40	76.25	13.07	62.66	7.31	67.30	9.78
BMI (Kg/m2)	25.15	4.67	29.79	3.00	28.53	3.48	23.16	1.86
Compliance (%)*	90.36	6.17	95.31	5.41	83.41	18.33	63.13	16.74
Events (nº)**	0.67	1.07	0.25	0.50	0.31	0.48	0.80	0.84

*CG*: control group; *IG*: intervention group; *M*: median; *SD*: standard deviation; *: calculated as a percentage of the total of 48 sessions; **: number of events (falls, hospitalization, exacerbation of previous illness) during the intervention

Barthel test scores ranged from 68 to 74 points (Table 3). The IG reduced their scores 5.45 points more than the CG after the intervention (95% CI, 1.82- 12.72). However, the percentage of compliance significantly modulated the evolution of the scores on this scale (F = 8.98, *p* = 0.005, ηp2 = 0.22), observing a certain depreciation in the scale associated with greater training compliance, and vice versa. After this adjustment, the estimated marginal means of Barthel behaved similarly, so a group effect (F = 0.84, *p* = 0.365, ηp2 = 0.03), or interaction time per group (F = 0.07, *p* = 0.796, ηp2 < 0.01), was ruled out. A repeated measures ANOVA with covariate observed a main pre-post intervention effect (F = 11.17, *p* = 0.002, ηp2 = 0.26), indicating a reduction in dependency scores for both groups.

**Table 3:** Barthel Index

		IC 95%			
	Group	Median	Minimum	Maximum	SD
Pre-intervention	CG	73.75	66.03	81.47	15.76
	IG	74.17	67.67	80.66	14.06
Post-intervention	CG	72.81	63.82	81.80	18.35
	IG	67.78	57.81	77.74	21.57
Change	CG	-0.94	-4.76	2.88	7.79
	IG	-6.39	-12.04	-0.74	12.22

*CG*: control group; *IG*: intervention group; *IC*: confidence interval; *SD*: standard deviation; Barthel index score from 0 to 100

The CG increased their times in the TUG test, while the IG reduced them after training, improving over the control by 7.43 s (95% CI, 3.28- 11.59). A repeated measures ANOVA confirmed the treatment per group interaction effect (F = 13.37, *p* < 0.001, ηp2 = 0.31), with a large effect size. Finally, no main effect was found for treatment per time effect (F = 0.07, *p* = 0.787, ηp2 < 0.01).

For the usual Gait Speed Test, participants took between 8 and 10 s to complete the 10 m. The IG reduced their time after training by 5.19 s (95% CI, 1.41- 8.97) with respect to the CG. A repeated measures ANOVA confirmed this large interaction effect between treatment per group (F = 9.41, *p* = 0.004, ηp2 = 0.23). No main effects were found by time (F = 1.00, *p* = 0.325, ηp2 = 0.03), nor by group (F = 0.02, *p* = 0.900, ηp2 < 0.01).

The scores for 10-MWT at maximum speed (Table 4) were between 7 and 9 s. As with the previous variable, the IG showed an improvement of 4.43 s (95% CI, 1.14–7.73) over the CG after training. A repeated measures ANOVA revealed a large treatment per group interaction effect, F = 11.66, *p* = 0.002, ηp2 = 0.27. No further main effects were found for time, F = 0.04, *p* = 0.843, ηp2 < 0.01, nor for group, F = 0.08 *p* = 0.786, ηp2 < 0.01.

**Table 4:** 10 Meters Walking Test

		IC 95%			
	Group	Median	Minimum	Maximum	SD
Pre-intervention	CG	8.53	7.09	9.97	2.85
	IG	10.20	7.69	12.72	5.44
Post-intervention	CG	9.76	8.11	11.41	3.26
	IG	7.79	6.64	8.93	2.47
Change	CG	1.23	-0.03	2.48	2.48
	IG	-2.41	-4.26	-0.57	4.00

*CG*: control group; *IG*: intervention group; *IC*: confidence interval; *SD*: standard deviation; 10 Meters Walking Test score in seconds

In the 6´MWT subjects covered a mean distance between 220 to 260 m. The IG showed an improvement of 54.54 m (95% CI, 30.24–78.84) over the CG after training. A repeated measures ANOVA confirmed the group interaction effect (F = 20.90, *p* < 0.001, ηp2 = 0.40), with a large effect size. No further main effects were revealed for pre-post intervention (F(1,32) = 0.11, *p* = 0.747, ηp2 < 0.01), or for group (F(1,32) = 0.92, *p* = 345, ηp2 = 0.03).

The SPPB scores ranged from 4 to 7 points. As with the 6´MWT, performance on the SPPB battery was strongly influenced by the 12-week intervention in the IG. After the multicomponent training program, the IG increased its performance by 2.74 points over the control (95% CI, 2.10- 3.37). Thus, a repeated measures ANOVA revealed a huge effect of the treatment per group (F = 77.96, *p* < 0.001, ηp2 = 0.71). Differences pre- post intervention (F = 2.46, *p* = 0.127, ηp2 = 0.07), and by group (F = 2.68, *p* = 0.112, ηp2 = 0.08), were also ruled out (Table 5). Moreover, the variables related to muscle maximum power and maximum strength did not show statistically significant differences in intergroup comparison.

**Table 5:** Short Physical Performance Battery

		IC 95%			
	Group	Median	Minimum	Maximum	SD
Pre-intervention	CG	5.44	4.44	6.43	2.03
	IG	5.33	4.26	6.41	2.33
Post-intervention	CG	4.31	3.30	5.32	2.06
	IG	6.94	5.72	8.17	2.65
Change	CG	-1.13	-1.59	-0.66	0.96
	IG	1.61	1.22	2.00	0.85

*CG*: control group; *IG*: intervention group; *IC*: confidence interval; *SD*: standard deviation; 10 Meters Walking Test score in seconds. SPPB score from 0 to 12

## Discussion

The results of this study are relevant when interpreted in the context of previous studies. Data collection post-intervention showed that in the TUG test participants in the IG improved their functionality and reduced their risk of falls by decreasing the time to perform the test by 7.43 s (*p* < 0.001). Many previous studies using the TUG test found improvements in the groups that performed exercise in different forms [23, 41, 42]. In terms of walking speed measured through the 10MWT, the IG improved their results by reducing the time to walk 10 m at usual speed by 5.19 s (*p* = 0.004), and maximum speed by 4.43 s (*p* = 0.002). Similar results regarding gait speed improvement were found by previous authors [34, 41–51]. Aerobic capacity, measured through the 6'WT improved in the IG (*p* < 0.001). Improvements in this capacity measured through the 6'WT, coincident with our analysis, were also observed in previous studies [41, 44, 48].

In relation to the SPPB, widely used to establish functional categories and detect frailty, the IG improved by + 2.74 points (*p* < 0.001). This result is important taking into account that the IG was only 0.06 points away from changing their frailty status to pre-frailty, as their post-intervention score was 6.94 points (the threshold to upgrade to pre-frailty is 7 points). Similar results were found in several studies with different exercise protocols [34, 42, 43, 45–47]. Losa-Reyna et al. [48] found similar improvements in SPPB results, through a shorter training protocol (6 weeks vs. 12 weeks), but with identical intervention characteristics, based on multicomponent training. It suggests that the response to multicomponent exercise is equally positive in frail older adults for gait speed, balance and functional mobility.

Trends were found, but were not statistically significant, in the Barthel index on autonomy in instrumental activities of daily living. These results are similar to those found by the studies of Martínez Velillaet al. [34] and Tarazona Santabalbina et al. [43] regarding the improvement of independence through exercise. The specific context of institutionalized patients (i.e., population with high dependence on these scales) could have conditioned the assessment of independence in this population. In this regard, we consider that the development of new tools to assess independence for activities of daily living in institutionalized patients are necessary for the correct assessment of this population group in their specific context.

Moreover, maximal strength and lower limb power were not significantly modified after the multicomponent exercise intervention period. Similar findings were shown in the review conducted by Haider et al. [47], where 3 studies found no improvements in muscle strength after different training programs, and 2 studies found improvements without statistically significant differences. These results could be due to a lack of capacity in the physiological response to the stimulus produced by participants’ training. Even when the fundamental variables of training (intensity, volume, individualization and compliance) are ensured, the response capacity of each individual is highly variable and can be modified very little.

The results obtained in study show that a multicomponent training, based on lower limb strength exercise and aerobic endurance, is safe and effective for improving frailty in institutionalized older adults. The protocol used in our research complies with some aspects recommended by the WHO regarding physical activity for older people with chronic conditions [12]. For instance, the strong recommendation, based on moderate certainty evidence, to add varied multicomponent physical activity at least three times a week, prioritizing functional balance and moderate or higher intensity strength training. In terms of the amount of physical activity, our protocol is close to the recommendation of doing at least 150 to 300 min of moderate physical activity, or between 75 and 150 min of vigorous physical activity, or an equivalent combination throughout the week. The protocol also fulfills the good practice statements of this document regarding the inclusion of physical activity that gradually increases in duration, frequency, and intensity.

In the same line, Mikel Izquierdo et al., in their publication on physical activity guidelines in older people published in 2021 [52], recommend individualized physical activity based on multicomponent physical exercise for improving health in older people. It includes the recommendation to continue investigating to know the most precise possible dose in terms of duration, volume, intensity, and type of exercise, as well as the inclusion of exercise as a fundamental piece, so far underused and with little penetration in institutions and health systems, for older adults.

Some limitations have to be acknowledged. The duration of the program (12 weeks) may not be as effective in an elderly population as it is in younger subjects, which could have biased some results. Nutrition and rest time were not recorded in this study. These variables can potentially have an influence on physical performance. Longer programs, monitoring nutrition and rest time, need to be tested in the institutionalized population in order to observe further physiological changes.

## Conclusion

A multicomponent exercise training program, based on lower limb resistance exercises and an aerobic exercise protocol, has been demonstrated to be safe and effective for the management of several relevant health indicators such as gait speed, functional mobility, functional capacity and balance in institutionalized older adults. The results also highlight the need for continued research to determine the optimal 'dose' of exercise, considering factors such as duration, volume, intensity, and type. It is important to acknowledge and integrate exercise as a vital, yet often underutilized component of older adults' healthcare, noting that it has not been sufficiently implemented in healthcare institutions and systems thus far.
