# Correction to: Functional mobility and physical fitness are improved through a multicomponent training program in institutionalized older adults

**DOI:** 10.1007/s11357-023-01026-7

**Published:** 2023-12-06

**Authors:** Sergio López‑López, Vanesa Abuín‑Porras, Luis A. Berlanga, Michelle Matos‑Duarte, Luis Perea‑Unceta, Carlos Romero‑Morales, Helios Pareja‑Galeano

**Affiliations:** 1 Faculty of Sport Sciences, Universidad Europea de Madrid, Madrid, Spain; 2 Department of Physical Activity and Sport, Centro de Estudios Universitarios Cardenal Spínola CEU, Seville, Spain; 3 Faculty of Health Sciences, Universidad Francisco de Vitoria, Madrid, Spain; 4 Department of Physical Education, Sport and Human Movement, Universidad Autónoma de Madrid, Madrid, Spain

**Correction to: GeroScience**

**https://doi.org/10.1007/s11357-023-00877-4**

The original version of this article unfortunately contained an error in the authorname of the 4th author. The correct authorname should have been Michelle Matos‑Duarte and it is already corrected in the authorgroup and affiliation section.

